# Pharmacokinetics of Cannabidiol in Rat Brain Tissue After Single-Dose Administration of Different Formulations

**DOI:** 10.3390/molecules30132676

**Published:** 2025-06-20

**Authors:** Zuzana Binova, Frantisek Benes, Marie Zlechovcova, Matej Maly, Petr Kastanek, Monika Cahova, Milena Stranska, Jana Hajslova

**Affiliations:** 1 Department of Food Analysis and Nutrition, University of Chemistry and Technology, Technická 3, 166 28 Prague, Czech Republic; zuzana.binova@vscht.cz (Z.B.); frantisek.benes@vscht.cz (F.B.); marie.zlechovcova@vscht.cz (M.Z.); matej.maly@vscht.cz (M.M.); milena.stranska@vscht.cz (M.S.); 2 Department of Biotechnology, University of Chemistry and Technology Prague, Technická 5, 166 28 Prague 6, Czech Republic; petr.kastanek@vscht.cz; 3 Institute for Clinical and Experimental Medicine, Vídeňská 1958, 140 21 Prague 4, Czech Republic; moca@ikem.cz

**Keywords:** Cannabidiol, metabolites, brain tissue, bioavailability, LC-MS/MS

## Abstract

Cannabidiol (CBD), a phytocannabinoid commonly isolated from chemotype III *Cannabis sativa* plants, is known for its therapeutic potential. However, comprehensive information on its bioavailability is still lacking. The key objective of this study was to investigate the impact of specific formulations on CBD delivery to the site of action and, in particular, the brain of experimental animals. As brain tissue is an extremely complex matrix, a highly sensitive method employing liquid chromatography–tandem mass spectrometry (LC-MS/MS) had to be implemented. To make it applicable for multiple analytes, the method was validated for 17 other phytocannabinoids and selected metabolites. Using this method, a pharmacokinetic study was conducted on 200 brain samples collected from rats that had been administered various CBD formulations (carriers) via oral gavage. The peak concentration in brain occurred within 1–2 h; notably, the highest was reached with carriers containing triacylglycerols with the shortest fatty acid chains (caprylic/capric). In addition to the parent compound, 7-hydroxy-cannabidiol and 7-carboxy-cannabidiol were detected, confirming rapid post-administration metabolism. Overall, this research enhances understanding of CBD distribution in the brain and underscores the impact of specific formulations on its bioavailability, offering insights into optimizing CBD-based therapies to be both effective and ‘patient-friendly’.

## 1. Introduction

Phytocannabinoids are unique biologically active secondary metabolites that occur in cannabis plants (*Cannabis sativa* L.). The best-known representatives are the psychotropic Δ^9^-tetrahydrocannabinol (Δ^9^-THC) and its non-psychotropic isomer cannabidiol (CBD). CBD is currently one of the most discussed phytocannabinoid molecules due to its promising therapeutic effects [1]. Various clinical studies have shown beneficial effects of CBD in the treatment of various health disorders, such as anxiety, epilepsy, or multiple sclerosis. In addition to CBD and THC, some other phytocannabinoids have been tested for their potential use in medicine. For example, cannabigerol (CBG), cannabinol (CBN), and cannabichromene (CBC) have been shown to have antibacterial effects, which have also been demonstrated for CBD and Δ^9^-THC. In addition, CBC shows analgesic effects, cannabidivarin (CBDV) anticonvulsant effects, CBG neuroprotective effects in experimental models of Huntington’s disease, and tetrahydrocannabivarin (THCV) improves insulin sensitivity and could be involved in the treatment of glucose intolerance associated with obesity [1,2].

The promising beneficial effects of phytocannabinoids on human health have led to their popularity among consumers, as evidenced by the wide range of available CBD products on the market. Besides food and dietary supplements (e.g., CBD oils), various ‘new medicines’ based on cannabis extracts or isolated phytocannabinoids have been advertised [1,2,3]. CBD can also be synthesized in a laboratory, allowing greater control over the purity and concentration of the final product [4]. From the user/patient point of view, oral administration of any supplement or medicine, including those with phytocannabinoids, is the most convenient way due to its independent application potential (without medical personnel) [5,6]. However, oral administration requires suitable dosage forms that ensure adequate bioavailability after ingestion. Moreover, the use of inappropriate dosage forms can lead to the ‘first pass effect’, where the liver eliminates the active compound before it reaches its intended site of action [6,7]. Due to the lipophilic nature, which limits the solubility of phytocannabinoids in aqueous solutions, they are often delivered in oil-based forms. However, gastrointestinal (GI) absorption of phytocannabinoids from oil, i.e., their bioavailability, is typically low [8]. Previous research, including animal studies, has indicated that oral consumption of CBD oils results in a maximum average bioavailability of 16% [9].

In recent years, efforts have been made to develop hydrophilic carriers chemically stable within the body, to enhance phytocannabinoids’ solubility in aqueous solutions [10]. One potential solution to this challenge is a nanoemulsification system, where immiscible liquids are combined into a suitable formulation using an emulsifier. This allows for easy oral administration of phytocannabinoids. However, these nanoemulsion systems are susceptible to digestion in the GI tract, which, in turn, can lead to precipitation and hindered absorption of the active compound if the system is chosen incorrectly [11]. Another approach to improve the solubility of hydrophobic compounds including phytocannabinoids is through a proliposomal formulation. Its lipid bilayer structure can optimize compound solubility and improve absorption by protecting the active ingredient from degradation in the gastrointestinal tract [12]. Several CBD preparations based on nanoemulsification systems (containing castor oil or medium-chain triacylglycerols) and proliposomes have been evaluated in pharmacokinetic studies in animal models, demonstrating their ability to improve CBD solubility, stability, and oral bioavailability. Other nanocarriers, such as polymeric micelles and lipid nanocapsules, provide sustained release and better targeting of CBD, leading to enhanced therapeutic efficacy [5,10,12].

Phytocannabinoids are primarily absorbed by tissues such as the heart, lung, liver, and brain, with the blood-to-tissue distribution occurring relatively rapidly. Understanding the pharmacokinetics of phytocannabinoids, especially in the brain, is particularly important because the brain contains the largest distribution of cannabinoid receptors and is the site of their pharmacological action. When assessing the efficacy of any bioactive compound and evaluating the suitability of its pharmaceutical dosing forms, parameters such as absorption, distribution, metabolism, and elimination must be closely monitored [13]. For this purpose, the availability of highly sensitive and specific analytical methods is imperative. Analyzing phytocannabinoids in brain tissue is a challenging task due to its inherent complexity, especially its high lipid content. Brain lipids (mainly phospholipids and cholesterol esters) play a fundamental role in the structure and function of this organ but can complicate the extraction and analysis of phytocannabinoids and interfere with their detection [14,15]. Various sorbents, mostly SPE (solid-phase extraction) cartridges, were used for the removal of co-isolated lipids from primary extracts. The number of studies quantifying cannabinoids in brain tissue is rather limited; moreover, CBD and/or THC and their metabolites were the only compounds studied [16,17,18,19,20,21]. However, due to the current emphasis on the therapeutic use of phytocannabinoids in the form of ‘full spectrum’ products rather than isolates, as well as the common presence of other phytocannabinoids in these so-called ‘pure’ CBD isolates (possibly affecting the overall CBD biological effect—depending on the dose), the widest possible spectrum of phytocannabinoids should be monitored not only in therapeutic products but also in pharmacokinetic studies [22,23].

The objectives of this study were, therefore, first to develop and validate a sensitive UHPLC-MS/MS (Ultra-high performance liquid chromatography coupled with tandem mass spectrometry) method for the determination of 18 phytocannabinoids, 3 Δ^9^-THC metabolites, and 2 CBD metabolites in brain tissue using a small sample for analysis; and second, to use this method to evaluate the pharmacokinetics of differently formulated CBD.

## 2. Results and Discussion

### 2.1. Optimization of Extraction Method

In general, brain tissue obtained from small animals (such as mice or rats) studies represent rare material of limited size, which might be required to be analyzed by more than one method targeting various analytes and using different approaches differing in the sample preparation (e.g., target analyses of specific compounds, metabolomics, and lipidomics). With this in mind, we aimed to develop a simple but effective extraction method that processes only a small sample size and also enables time- and cost-feasible analysis of such a large sample set, as is the case in our CBD (cannabidiol) pharmacokinetic study.

As a first step, four different solvent mixtures (acetonitrile, methanol, ethanol, and isopropyl alcohol) previously used in similar studies [24,25,26] were tested to isolate phytocannabinoids from spiked rat brain tissue. The best extraction efficiency was achieved using acetonitrile. Subsequently, various modifications of the acetonitrile-based extraction procedure were tested (acetonitrile/water ratios tested were 3:1 and 30:1, *v*/*v*). The best extraction efficiency assessed for major phytocannabinoids is presented in Supplementary Table S1. Due to high chemical noise in the analysis of these primary extracts, a modified QuEChERS-like (Quick, Easy, Cheap, Effective, Rugged, and Safe) extraction [27], based on the separation of aqueous and organic (acetonitrile) phases by the addition of inorganic salts, was employed. The analysis of the obtained acetonitrile phase showed a decrease in matrix co-extracts; therefore, no further clean-up was performed. For most analytes, good performance characteristics were achieved in terms of recovery, repeatability, and sufficient removal of interferences from the acetonitrile extract. The values of individual parameters of the developed method employing QuEChERS extraction are discussed in Section 2.3.

Most previously published methods for phytocannabinoid analysis in brain tissue [16,17,18,19,21,25,26] used relatively large samples (usually hundreds of milligrams to grams), and various purification steps based on SPE (solid phase extraction) had to be used prior to LC-MS analysis. Thanks to the selectivity of QuEChERS extraction and the very small sample size (40 mg) taken for analysis, no further cleanup step was needed in our procedure employing a triple quadrupole mass spectrometer for target analyte detection.

### 2.2. Development and Optimization of the UHPLC-MS/MS Method

When analyzing multiple phytocannabinoids and their metabolites, i.e., analytes occurring at trace levels in a complex matrix such as brain tissue, ultra-high performance liquid chromatography coupled with mass spectrometry, employing either a tandem low-resolution mass analyzer or, when available, high-resolution mass analyzers, is the technique of choice [26]. For the purpose of target analysis, a UHPLC-MS/MS (Ultra-high performance liquid chromatography coupled with tandem mass spectrometry) system equipped with a triple-quadrupole mass analyzer was employed. The aim was to develop a multidetection method including not only CBD and its main metabolites expected to be present in brain tissue samples, but also other phytocannabinoids for which analytical standards were available at that time in our laboratory. Such a method would then be ready to use for any future pharmacokinetic studies of other phytocannabinoids or full-spectrum cannabis extracts.

First, the MS method parameters were carefully tuned to get optimal transition for each analyte, including the ion source parameters (according to Supplementary Table S2), and a complete summary of the final settings is given in Section 3.7.

**Figure 1:**
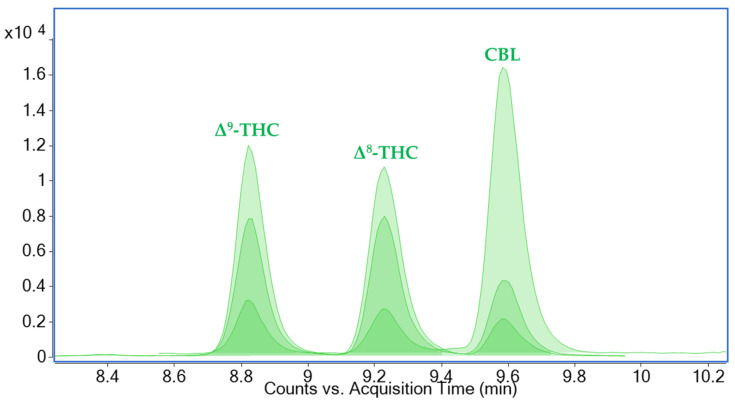
A dynamic multiple-reaction-monitoring (dMRM) ion chromatogram showing specific precursor-to-product ion transitions and their relative ratios for the isomeric neutral phytocannabinoids Δ^9^-THC (Δ^9^-tetrahydrocannabinol), Δ^8^-THC (Δ^8^-tetrahydrocannabinol), and CBL (cannabicyclol) (*m*/*z* 315.2) in brain tissue extract. The concentration of each analyte was 80 µg/kg. UHPLC-MS/MS (Ultra-high performance liquid chromatography coupled with tandem mass spectrometry) conditions are described in Section 3.7.

**Figure 2:**
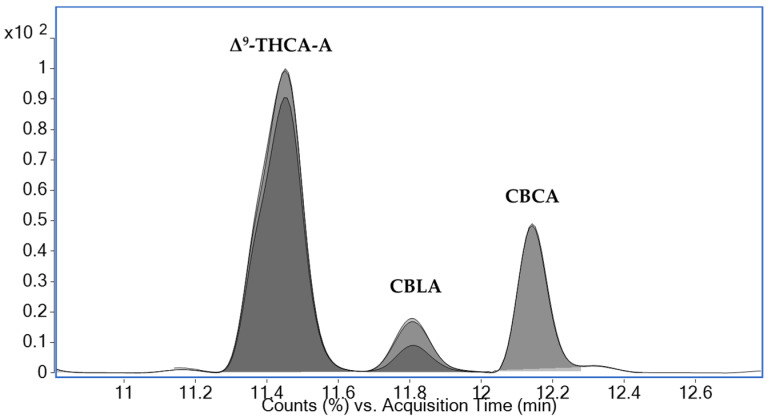
A dynamic multiple-reaction-monitoring (dMRM) ion chromatogram showing specific precursor-to-product ion transitions and their relative ratios for the isomeric phytocannabinoid acids Δ^9^-THCA-A (Δ^9^-tetrahydrocannabinolic acid A), CBLA (cannabicyclolic acid), and CBCA (cannabichromenic acid) (*m*/*z* 357.2) in brain tissue extract. The concentration of each analyte was 80 µg/kg. UHPLC-MS/MS (Ultra-high performance liquid chromatography coupled with tandem mass spectrometry) conditions are described in Section 3.7.

The optimization of chromatographic separation of target compounds on a reversed-phase column was performed by using various compositions of mobile phases and gradient elution. As some of the phytocannabinoids are isomeric compounds that provide identical fragmentation spectra, their sufficient chromatographic separation is crucial for their reliable identification; the critical isomers with similar retention behavior on reverse column are especially Δ^9^-tetrahydrocannabinol (Δ^9^-THC), Δ^8^-tetrahydrocannabinol (Δ^8^-THC), cannabichromene (CBC), and cannabicyclol (CBL), and their respective acids. In addition to the solvent composition and gradient method described in Section 3.7, the separation of phytocannabinoids, including critical isomers (especially cannabichromenic acid (CBCA) and cannabicyclolic acid (CBLA)), was influenced by the addition of different mobile phase modifiers. The co-elution of CBCA and CBLA occurred when only formic acid (0.1%) was added, while subsequent addition of 5 mM ammonium formate caused their complete separation. When the concentration of formic acid was increased (up to 0.3%), higher signal intensities were observed for all analytes, but the coelution of CBCA and CBLA occurred again. The use of acetic acid and ammonium acetate led to an overall reduction in the intensity of the signal compared to the combination of formic acid and ammonium formate, especially in the case of analytes detected in positive electrospray ionization (ESI^+^). Thus, 5 mM ammonium formate and 0.1% formic acid were chosen as final modifiers, providing the best peak shape and separation of neutral phytocannabinoids and their acids. The successful separation of the main critical isomers is illustrated in Figure 1 and Figure 2. Under the final optimized conditions, all eighteen analytes and five metabolites were eluted within 12 min, followed by the application of 100% organic mobile phase to ensure the removal of other matrix coextracts with high retention factors, such as phospholipids, triacylglycerols, and cholesterol esters.

The sample injection volume in the 1 to 5 µL range was also evaluated. The optimal peak shapes were achieved at an injection volume of 1 µL, a setting that minimized matrix interferences’ introduction into the instrumental system.

**Figure 3:**
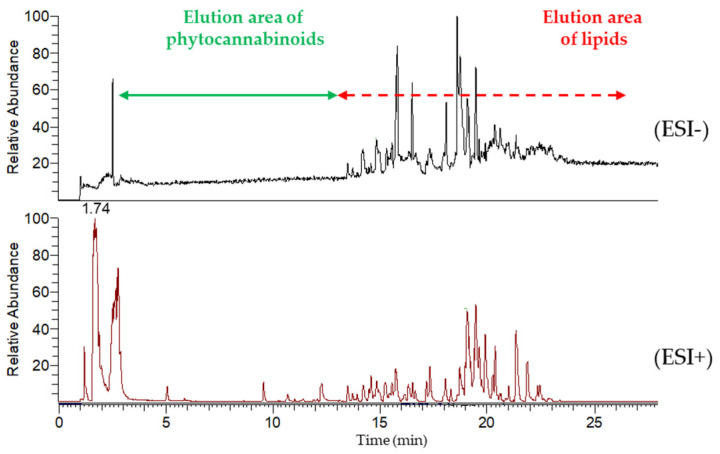
Total ion chromatograms (TICs) of blank rat brain matrix in negative (ESI^-^) and positive (ESI^+^) ionization modes (full MS 100–1200 *m*/*z*). UHPLC-HRMS/MS (Ultra-high performance liquid chromatography coupled with tandem mass spectrometry) conditions are described in Section 3.8.

Non-target screening by high-resolution mass spectrometry was employed for a more in-depth investigation of UHPLC-separated matrix components contained in brain extract. The Q-Exactive Plus Hybrid Quadrupole-Orbitrap mass spectrometer was operated under the conditions described in Section 3.8. Chromatograms in Figure 3 illustrate the retention time range in which target phytocannabinoids elute, as well as the elution of various lipids. While in negative ionization mode mostly phospholipids are detected, later-eluting, less polar triacylglycerols are detected mainly in positive mode as ammonium adducts. In any case, the complexity of the brain matrix is documented here. As far as the UHPLC run is not long enough, several matrix effects would occur including suppression/enhancement of ionization and shifts in retention times if other matrix components (phospholipids, triacylglycerols, or cholesterol) were not washed out of the chromatography column after separation of phytocannabinoids.

### 2.3. Validation of the Method

The target UHPLC-MS/MS method was carefully validated according to the International Council for Harmonisation of Technical Requirements for Pharmaceuticals for Human Use (ICH) Q2(R2) Validation Guidelines (2023) [28]. The suitability of the achieved performance for the given purpose was confirmed, thereby ensuring its reliability for the intended analytical applications.

#### 2.3.1. Homogeneity of Brain Tissue Samples

Verification of sufficient sample size (40 mg) for analysis and homogeneity of the test material was performed by analyzing the CBD content in the two randomly selected real rat brain tissue samples (each analyzed in six replicates). The inter-assay variability of the results expressed as relative standard deviation (RSD) was 15% for sample one and 17% for sample two.

#### 2.3.2. Limit of Quantification (LOQ), Linearity, Recovery, and Repeatability

The LOQ value (the lowest point of the calibration curve) for most neutral phytocannabinoids and their acids was 1.6 µg/kg brain tissue, except for cannabigerovarin (CBGV), CBD, CBC, cannabidivarinic acid (CBDVA), tetrahydrocannabivarinic acid (THCVA), and CBCA whose LOQ was 4 µg/kg. The LOQ for the metabolites (±)-11-hydroxy-Δ^9^-tetrahydrocannabinol (11-OH-Δ^9^-THC) and (+)-11-nor-9-carboxy-Δ^9^-tetrahydrocannabinol glucuronide (11-nor-9-COOH-Δ^9^-THC-glu) was 4 µg/kg and for (+)-11-nor-9-carboxy-Δ^9^-tetrahydrocannabinol (11-nor-9-COOH-Δ^9^-THC), 7-carboxy-cannabidiol (7-COOH-CBD), and 7-hydroxy-cannabidiol (7-OH-CBD) was 8 µg/kg brain tissue. Regarding the usual dosing of phytocannabinoids used in pharmacokinetic studies and the expected metabolism rate (based on other published studies [9]), the LOQ values were considered satisfactory for the sensitive detection of phytocannabinoids and their metabolites. The linearity of the method was determined in the presence of a matrix with a regression coefficient (*R*^2^) greater than 0.99 for all analytes in the range 1.6–400 µg/kg for phytocannabinoids and their acids and 4–400 µg/kg for phytocannabinoid metabolites. Mean recoveries ranged from 71–110% for neutral phytocannabinoids, 67–102% for phytocannabinoid acids, and 61–112% for metabolites. The method had good repeatability with RSD values of 1–12% for neutral phytocannabinoids, 2–14% for phytocannabinoid acids, and 1–20% for metabolites at all three concentration levels of 8, 80, and 160 µg/kg.

#### 2.3.3. Matrix Effects

As shown in Figure 3, phytocannabinoids elute before lipids, which are the major interferents in brain extracts. However, many other matrix co-isolates elute in the same retention time range as the target analytes and may cause matrix effects. Rat brain tissue spiked with a mixed solution of all target analytes at a concentration of 8, 80, and 160 µg/kg was analyzed together with the corresponding standards dissolved at the same concentrations in acetonitrile. The signals of all target analytes detected were then compared to evaluate possible matrix effects causing analyte signal suppression/enhancement (SSE %). Of the eighteen phytocannabinoids and five metabolites included, only five (cannabinolic acid (CBNA), CBC, Δ^9^-tetrahydrocannabinolic acid A (Δ^9^-THCA-A), cannabicyclolic acid (CBLA), and cannabichromenic acid (CBCA)) were notably affected by matrix effects, with SSE values ranging from 150–290% for each concentration level. As these analytes are eluted from the chromatographic column within the last 2 min of the gradient composed of a high percentage of the organic mobile phase, the presence of the co-eluted matrix interferences is not surprising. These results highlight the need for the use of matrix-matched calibration or internal standard correction to achieve reliable quantification for analytes affected by matrix effects.

The detailed overview of the method validation parameters and the SSE values for all analytes is presented in Supplementary Table S3. Despite a considerably wider range of included analytes, our method achieves comparable values of the validation parameters, especially LOQs, precision, and repeatability, compared to previously published methods [16,17,18,19,20,21,24,25]. This demonstrates the robustness and efficiency of our method in quantifying a wide spectrum of phytocannabinoids and their metabolites in brain tissue.

### 2.4. Transfer of CBD into the Brain of Exposed Animals

The developed and validated method for the targeted analysis of phytocannabinoids was applied to the analysis of a set of rat brain tissue samples from the CBD pharmacokinetic study. Average concentrations of CBD and its metabolites were calculated for each of the sampling time points (Table 1, based on data from four to six independent observations per time point). The following CBD pharmacokinetic parameters (PK) were then assessed: the maximum brain concentration (*C*_max_) over time (up to 6 h after CBD administration), the time needed to reach maximum concentration (*T*_max_) in brain tissue, and biotransformation. The area under the curve (AUC_0–6_) illustrating changes in CBD concentration in brain tissue over 6 h period was used to assess the total animal’s body exposure by CBD (linear trapezoidal rule from time 0 to time of the last quantifiable concentration employed for area calculation [29]). All PK values are summarized in Table 2. Notably, there were no negative impacts on locomotor behavior and cognitive performance in the studied animals after CBD administration.

#### 2.4.1. Pharmacokinetic Findings of CBD

**Figure 4:**
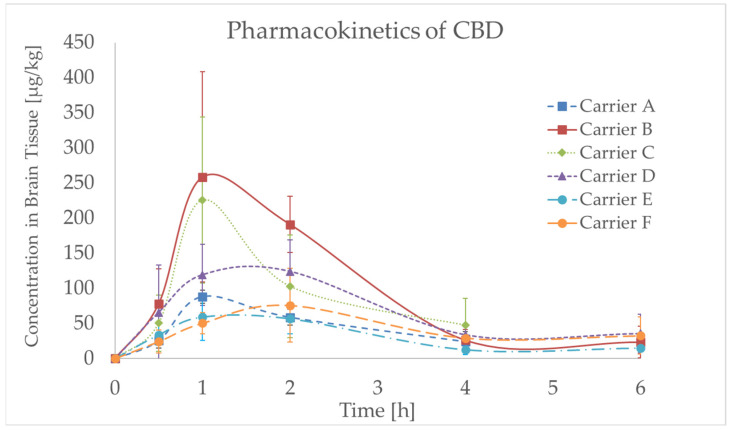
Concentration–time profile for CBD (cannabidiol) in rat brain tissue after 10 mg/kg oral gavage administration of various CBD carriers, including CBD powder (Carrier A), CBD oil in caprylic/capric triacylglycerols (Carrier B), self-emulsifying drug delivery system (SEDDS) with MCT (Medium-Chain Triglyceride) oil (Carrier C) or sesame oil (Carrier D), and CBD proliposomes with chitosan coating, either in crystalline NUTRIOSE form (Carrier E) or prepared by spray drying (Carrier F); data are expressed as mean ± standard deviation (SD) (*n* = 4–6 per time point).

Figure 4 shows a rapid increase in CBD in brain tissue for all tested carriers; the maximum concentration, *C*_max_, was reached within 1–2 h (*T*_max_) after gavage administration; then, a slower decrease occurred until 4 h, and no notable changes were observed between 4 h and 6 h. The declared CBD content in each formulation was analytically confirmed prior to administration, as described in Section 3.6. The *T*_max_ value complies with the findings of other similar studies. In the review by Millar et al. (2018), which documented the dose dependence of *C*_max_, reported *T*_max_ generally ranged between 1 and 4 h [9]. As longer measurements were not performed and matrices other than the brain were not available, a more detailed description of CBD distribution in the animals’ bodies and the way of excretion could not be obtained.

In our single-dose study (10 mg CBD/kg for all animals), the highest mean *C*_max_ of CBD in the brain was 259 ± 150 µg/kg, recorded 1 h after administration of carrier B (10% CBD oil). In this case, the highest AUC_0–6_ (596 µg/kg × h) was calculated, indicating the highest exposure of brain tissue by CBD. Conversely, the lowest *C*_max_ of 59 ± 33 µg/kg and correspondingly relatively low AUC_0–6_ (186 µg/kg × h) were observed for carrier E (CBD proliposomes-CC).

As shown in Figure 4 and Table 2, the pharmacokinetic profile of CBD in brain tissue was strongly influenced by the formulation used. The shortest *T*_max_ (1 h) was observed for carriers B (10% CBD oil) and C (SEDDS with MCT (Medium-Chain Triglyceride) oil), which also achieved the highest *C*_max_ values (259 and 226 µg/kg) and AUC_0–6_ values (596 and 397 µg/kg x h), indicating rapid and efficient brain delivery. These results suggest that formulations promoting fast solubilization and absorption (such as oil-based and MCT-based SEDDS) enhance CBD uptake into brain tissue.

On the other hand, carriers D (SEDDS with sesame oil) and F (proliposomes—spray drying) reached *T*_max_ later (2 h), yet still achieved relatively high AUC_0–6_ values (412 and 255 µg/kg x h), pointing to a more sustained CBD release. Interestingly, although their *C*_max_ values were lower compared to B and C, the prolonged exposure time may be beneficial in maintaining CBD levels over time. Carrier E (proliposomes with chitosan coating) showed both low *C*_max_ (59 µg/kg) and AUC_0–6_ (186 µg/kg x h), indicating less efficient delivery to brain tissue.

These findings highlight that a delayed *T*_max_ does not necessarily indicate poor performance; on the contrary, it may reflect sustained drug release, which can be advantageous depending on the therapeutic context. In summary, carriers A, D, E, and F showed a slower decrease in CBD concentration over time compared to carriers B (10% CBD oil) and C (SEDDS with MCT oil) which might be desirable for therapeutic purposes.

The impact of the type of oil used in the CBD-formulated SEDDS on *C*_max_ was also assessed. Higher *C*_max_ (226 ± 118 µg/kg) was achieved by carrier C (SEDDS with MCT oil) compared to carrier D (SEDDS with sesame oil) for which the *C*_max_ was 124 ± 45 µg/kg. Interestingly, the highest *C*_max_ was achieved when triacylglycerols with the shortest fatty acid chains (caprylic/capric, C8:0/C10:0, in the case of carrier B) were used for CBD formulation. Generally, an increase in the molecular weight of triacylglycerols used for this purpose resulted in a decrease in *C*_max_. MCT oil (carrier C) contains medium-chain fatty acids such as lauric acid (C12:0) and myristic acid (C14:0), while sesame oil (carrier D) predominantly contains long-chain fatty acids such as linoleic acid (C18:2) and oleic acid (C18:1). While the influence of excipients on CBD pharmacokinetics has been explored in previous studies, research specifically addressing its distribution in the brain remains limited. Nevertheless, the dependence of CBD intestinal lymphatic transport and systemic bioavailability on the composition of vegetable oils as a delivery system has been documented in a recent study [30].

A more detailed comparison of the PK parameters reveals that oil-based and MCT-based carriers (B and C) result in faster absorption and higher brain exposure, as reflected by elevated AUC_0–6_ values and *C*_max_. In contrast, proliposomal systems (carriers E and F) show a slower onset and lower peak levels, suggesting a sustained-release effect. Such differences underscore the importance of formulation type in modulating CBD pharmacokinetics and brain availability, which is critical for developing targeted CNS (central nervous system) delivery strategies.

Unfortunately, plasma data were not collected at this stage, as the primary objective of the present study was to focus on brain pharmacokinetics. This limitation is acknowledged, and future work will include plasma profiling and release studies of CBD formulations for a better understanding of formulation effects on CBD pharmacokinetics.

#### 2.4.2. Pharmacokinetic Findings of CBD Metabolites

**Figure 5:**
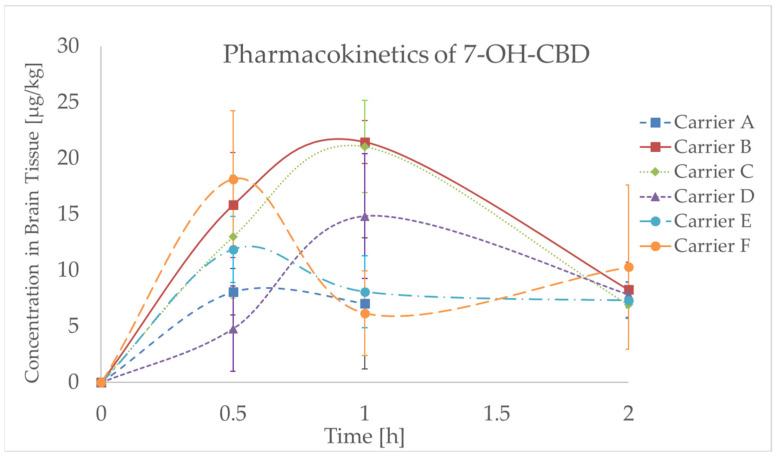
Concentration–time profile for 7-OH-CBD (7-hydroxycannabidiol) in rat brain tissue after 10 mg/kg oral gavage administration of various CBD (cannabidiol) carriers, including CBD powder (Carrier A), CBD oil in caprylic/capric triacylglycerols (Carrier B), self-emulsifying drug delivery system (SEDDS) with MCT (Medium-Chain Triglyceride) oil (Carrier C) or sesame oil (Carrier D), and CBD proliposomes with chitosan coating, either in crystalline NUTRIOSE form (Carrier E) or prepared by spray drying (Carrier F); data are expressed as mean ± standard deviation (SD) (*n* = 4–6 per time point).

**Figure 6:**
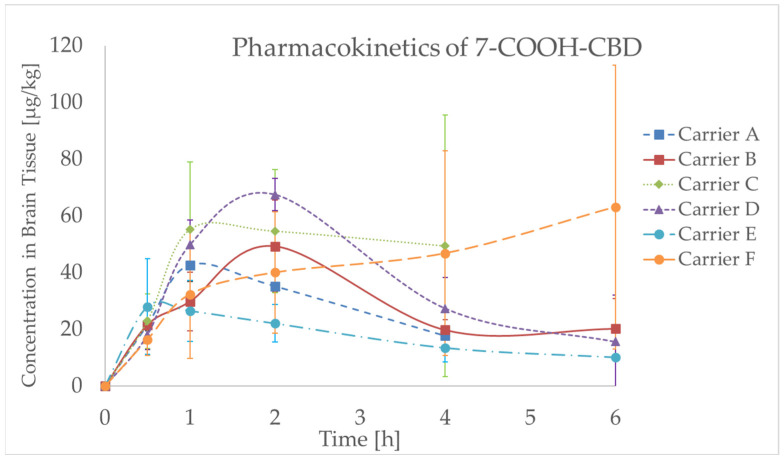
Concentration–time profile for 7-COOH-CBD (7-carboxycannabidiol) in rat brain tissue after 10 mg/kg oral gavage administration of various CBD (cannabidiol) carriers, including CBD powder (Carrier A), CBD oil in caprylic/capric triacylglycerols (Carrier B), self-emulsifying drug delivery system (SEDDS) with MCT (Medium-Chain Triglyceride) oil (Carrier C) or sesame oil (Carrier D), and CBD proliposomes with chitosan coating, either in crystalline NUTRIOSE form (Carrier E) or prepared by spray drying (Carrier F); data are expressed as mean ± standard deviation (SD) (*n* = 4–6 per time point).

In the next phase, two major phase I CBD metabolites originating through processes catalyzed by cytochrome P450 enzymes were monitored. While the primary metabolite, 7-hydroxycannabidiol (7-OH-CBD), is pharmacologically active, 7-carboxycannabidiol (7-COOH-CBD) does not exhibit such activity anymore [31,32]. Like in the case of CBD, the time-dependent profile of these metabolites differed depending on the carrier system used. For carrier C, the *T*_max_ values of both metabolites were identical to that of CBD (1 h). Shorter *T*_max_ values of 7-OH-CBD were observed for carriers A, D, E, and F (0.5, 1, 0.5, and 1 h, respectively). For carrier B, the *T*_max_ of 7-COOH-CBD was delayed to 2 h, whereas for carrier F, it was substantially prolonged to 6 h, compared to 2 h for CBD. This delay, together with a relatively high AUC_0–6_, may be linked to slower CBD release from proliposomal matrices and high inter-individual variability at later time points. The measured data are illustrated in Figure 5 and Figure 6 and summarized in Table 2.

In some cases, the concentration–time curves appeared biphasic, with a secondary rise. This pattern may reflect formulation-dependent absorption, delayed release, or redistribution mechanisms [33].

**Figure 7:**
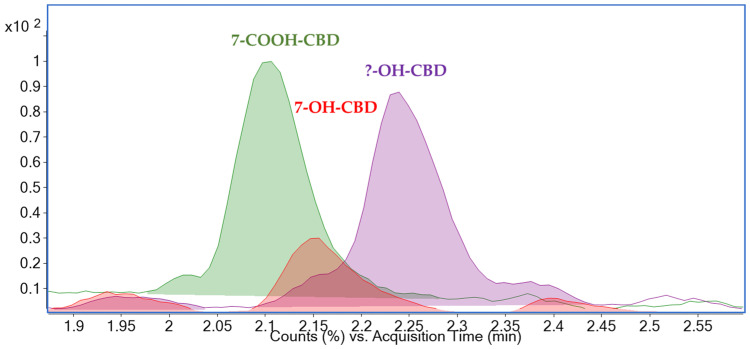
Chromatogram of target metabolites (precursor ion *m*/*z* 345.2, 7-COOH-CBD—green (7-carboxycannabidiol); *m*/*z* 331.2, 7-OH-CBD—red (7-hydroxycannabidiol)) and an unknown metabolite (precursor ion *m*/*z* 331.2—purple) in a rat brain extract (vehicle A). The determined concentrations of 7-COOH-CBD and 7-OH-CBD were 22.2 µg/kg and 6.9 µg/kg, respectively. The unknown metabolite (purple) was detected but not quantified. UHPLC-MS/MS (Ultra-high performance liquid chromatography coupled with tandem mass spectrometry) conditions are described in Section 3.7.

In the final phase of CBD metabolites investigation, we inspected carefully the records of the targeted UHPLC-MS/MS analysis and in each of them, the presence of an additional peak with identical dMRM (dynamic multiple-reaction-monitoring) transitions to those of 7-OH-CBD was found (see Figure 7). Regarding the identification of the ‘unknown’ peak, this was rather complicated when using MS with a triple quadrupole mass analyzer. Therefore, high-resolution mass spectrometry (HRMS) was employed to obtain more structural information. Nonetheless, the detected compound is likely one of the 7-OH-CBD isomers formed during CBD metabolism. Several studies have also reported signals corresponding to monohydroxylated CBD metabolites [25,34].

**Figure 8:**
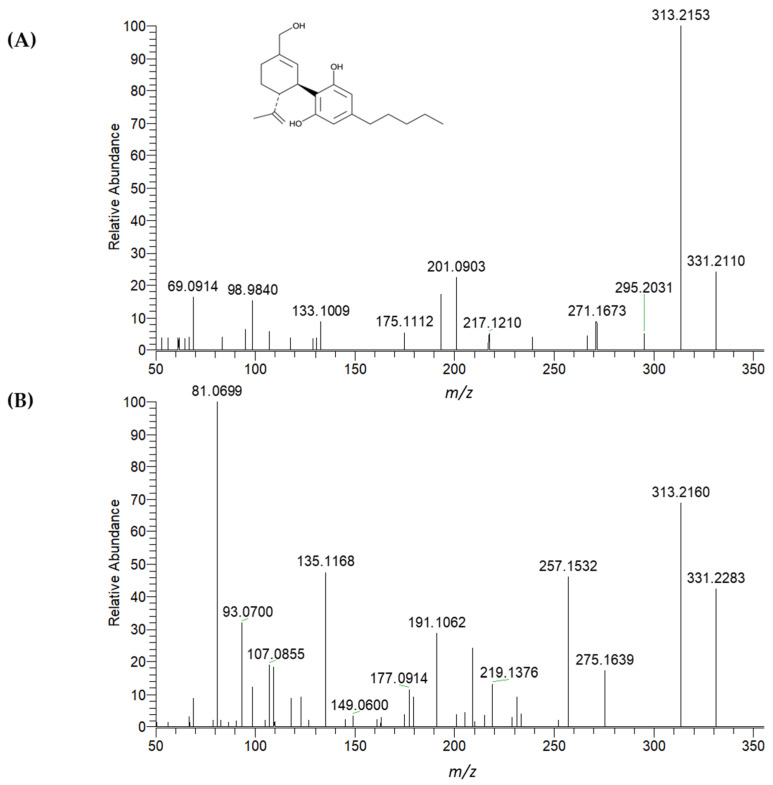
Experimentally obtained high-resolution fragmentation spectra of the protonated ion of 7-OH-CBD (7-hydroxycannabidiol) (**A**) and a tentatively identified hydroxylated metabolite (**B**) in ESI+ mode (*m*/*z* 331.2268). The spectra were acquired under identical conditions using UHPLC (Ultra-high performance liquid chromatography) with a BEH C18 column (150 × 2.1 mm; 1.7 µm), coupled to a Q-Exactive^TM^ Hybrid Quadrupole-Orbitrap^TM^ mass spectrometer. Separation was performed using a gradient elution with mobile phase (**A**) water/acetonitrile (95:5, *v*/*v*) and (**B**) methanol/acetonitrile/water (60:35:5, *v*/*v*/*v*), both containing 5 mM ammonium formate and 0.1% formic acid.

For further investigation, the UHPLC-HRMS/MS method described in Section 3.8 was used. The fragmentation spectra of 7-OH-CBD and the ‘unknown’ peak shown in Figure 8 were not identical; nevertheless, they appear to be isomers (C_21_H_30_O_3_). Although no database match was found for either spectrum, the protonated molecular ion (theoretical *m*/*z* 331.2268) and ion corresponding to a loss of water (theoretical *m*/*z* 313.2162) were practically identical.

Interestingly, the same ‘unknown’ metabolite was also detected in the study by Citti et al., along with other unidentified compounds with molecular ions corresponding to carboxy CBD or dihydroxylated CBD. It is worth noting that the CBD dose used in our study was five times lower than in the study by Citti et al., which may explain the absence of these minor metabolites in our results [25].

#### 2.4.3. Pharmacokinetic Variability

Variability among rats (*n* = 4–6) in the same testing group, i.e., administered by the same CBD formulation and euthanized after the same period of time, has been observed. This is probably due to the different morphology and functionality of their digestive tract, which may affect the absorption and distribution of the active compound among individual animals. The intragroup variability may also be an important criterion for assessing the efficacy of the drug in different individuals. The use of suitable formulation techniques can reduce this variability [35]. The least variability was observed with carrier A (CBD powder), which was not formulated in any specific way. This variability was reflected in relatively high standard deviations in some pharmacokinetic data.

Data were statistically processed using one-way analysis of variance (ANOVA) followed by post hoc Tukey’s range test using MetaboAnalyst 5.0 (https://www.metaboanalyst.ca/). However, due to the large variability among experimental animals, it cannot be claimed that there were statistically significant differences between the tested CBD formulations/carriers (*p*-value < 0.05).

## 3. Materials and Methods

### 3.1. Samples

A large collection of brain tissue samples (*n* = 200) from experimental animals was available for CBD bioavailability research. Wistar RCC Hahn rats with a weight range of 250–300 g were subjected to a single gavage administration of variously formulated CBD preparation (98% CBD isolate) at a dose of 10 mg CBD/kg rat body weight. In all cases, the total volume of the bolus was 1 mL. A total of 6 different CBD carriers (CBD formulations described below) were tested. Animals were euthanized at various predetermined time intervals (0, 0.5, 1, 2, 4, or 6 h) after administration of the CBD preparation by anesthetic overdose. Subsequently, brain tissue (~0.5 g) was collected, quickly frozen, and stored at −80 °C until UHPLC-MS/MS analysis. A ‘blank’ sample was obtained in the same way from the same animal experiment, except the animals were not given CBD.

#### CBD Formulations

- Carrier A (CBD powder): CBD standard powder (suspended in xanthan gum), i.e., CBD isolate 98%
- Carrier B (10% CBD oil): CBD oil (caprylic/capric triacylglycerols) and CBD: 10% (*w*/*w*)
- Carrier C (SEDDS with MCT oil): self-emulsifying drug delivery system (SEDDS) with medium-chain triacylglycerols (MCT) as carrier and CBD: 5% (*w*/*w*)
- Carrier D (SEDDS with Sesame Oil): self-emulsifying drug delivery system (SEDDS) with sesame oil as carrier and CBD: 2% (*w*/*w*)
- Carrier E (CBD Proliposomes-CC): CBD proliposomes with chitosan coating (CC), crystalline NUTRIOSE as a carrier and CBD: 4% (*w*/*w*)
- Carrier F (CBD Proliposomes-CC- Spray Drying): CBD proliposomes with chitosan coating (CC) prepared by spray drying, crystalline NUTRIOSE as a carrier and CBD: 4% (*w*/*w*)

The study was conducted in accordance with the Animal Protection Law of the Czech Republic No. 311/1997, which complies with the NIH Guide for the Care and Use of Laboratory Animals (8th edition, 2013). All procedures were approved by the Ethics Committee of the Institute for Clinical and Experimental Medicine and by the Ministry of Health of the Czech Republic (protocol code MZDR 40842/2018-4/OVZ, approved on 26 September 2018). Rearing rooms were maintained on a 12 h light–dark cycle under controlled conditions of temperature (22 ± 1 °C) and relative humidity (45–55%). Food and water were available ad libitum. The CBD formulations were prepared by EcoFuel laboratories s.r.o., using 98% CBD isolate powder.

### 3.2. Chemicals

Anhydrous magnesium sulfate and LC-MS-grade chemicals including methanol, acetonitrile, ethanol, isopropyl alcohol, ammonium formate and formic acid were purchased from Merck (Darmstadt, Germany). Deionized water with a resistance of 18 mΩ was generated using an internal Milli-Q^®^ system (Merck Millipore, Bedford, MA, USA). Sodium chloride was purchased from Penta s.r.o. (Prague, Czech Republic). Analytical standards of 18 phytocannabinoids, their metabolites and deuterated internal standards, namely cannabidiol (CBD), cannabidiolic acid (CBDA), Δ^9^- and Δ^8^-tetrahydrocannabinol (Δ^9^- and Δ^8^-THC), Δ^9^-tetrahydrocannabinolic acid A (Δ^9^-THCA-A), Δ^9^-tetrahydrocannabivarin (Δ^9^-THCV), tetrahydrocannabivarinic acid (THCVA), cannabinol (CBN), cannabinolic acid (CBNA), cannabigerol (CBG), cannabigerolic acid (CBGA), cannabidivarin (CBDV), cannabidivarinic acid (CBDVA), cannabichromene (CBC), cannabichromenic acid (CBCA), cannabicyclol (CBL), cannabicyclolic acid (CBLA), cannabigerovarine (CBGV), (±)-11-hydroxy-Δ^9^-tetrahydrocannabinol (11-OH-Δ^9^-THC), (+)-11-nor-9-carboxy-Δ^9^-tetrahydrocannabinol glucuronide (11-nor-9-COOH-Δ^9^-THC-glu), (+)-11-nor-9-carboxy-Δ^9^-tetrahydrocannabinol (11-nor-9-COOH-Δ^9^-THC), 7-carboxy-cannabidiol (7-COOH-CBD) and 7-hydroxy-cannabidiol (7-OH-CBD), cannabidiol-D_3_ (CBD-D_3_), Δ^9^-tetrahydrocannabinol-D_3_ (Δ^9^-THC-D_3_), cannabigerol-D_3_ (CBG-D_3_), cannabinol-D_3_ (CBN-D_3_), Δ^9^-tetrahydrocannabinol A acid D_3_ (Δ^9^-THCA-A-D_3_), 11-hydroxy-Δ^9^-tetrahydrocannabinol-D_3_ (11-OH-Δ^9^-THC-D_3_), 11-nor-9-carboxy-Δ^9^-tetrahydrocannabinol-D_9_ (11-nor-9-COOH-Δ^9^-THC-D_9_), 11-nor-9-carboxy-Δ^9^-tetrahydrocannabinol-glucuronide-D_3_ (11-nor-9-COOH-Δ^9^-THC-glu-D_3_), 7-hydroxy-cannabidiol-D_3_ (7-OH-CBD-D_3_), 7-carboxy-cannabidiol-D_3_ (7-COOH-CBD-D_3_), with the purity ranging from 98.3–99.8%, were obtained from Cerilliant Corporation (Round Rock, TX, USA) and Cayman Chemical (Ann Arbor, MI, USA). Phytocannabinoid acid standards were obtained as acetonitrile stock solutions, while other standards were obtained as methanolic stock solutions at a concentration of 1 mg/mL, except for cannabicyclic acid, which was at a concentration of 0.5 mg/mL. Metabolite and deuterated internal standards (ISTD) were obtained at a concentration of 0.1 mg/mL.

### 3.3. Preparation of Standard Solutions

A standard mixture with a concentration of 10,000 ng/mL of each compound was prepared from the stock solutions of all phytocannabinoids and metabolites and then serially diluted with acetonitrile to achieve concentrations of 1000, 100, 10, and 1 ng/mL. These mixtures of standards were used for the preparation of the matrix-matched calibration standards and artificially enriched (spiked) samples.

An acetonitrile-based ISTD mixture (300 ng/mL per compound) was prepared from stock solutions. All solutions were stored at −18 °C.

### 3.4. Sample Preparation

#### 3.4.1. Brain Tissue

The entire sample of each collected brain tissue was carefully homogenized with liquid nitrogen (SIAD Czech spol. s r.o., Prague, Czech Republic). Subsequently, 40 mg was weighed in a 1.5 mL plastic Eppendorf tube (Eppendorf AG, Hamburg, Germany). A total of 30 μL of deionized water acidified with 0.1% formic acid and 80 μL of acetonitrile spiked with a mixture of ISTDs were added to the sample. The contents of the Eppendorf tube were thoroughly mixed for 1 min using a Vortex Mixer homogenizer (LACHOI Scientific Instrument Co., Ltd., Shaoxing, China). Subsequently, 40 mg of magnesium sulfate and 8 mg of sodium chloride were added to the tube and its content was mixed again. The resulting mixture was centrifuged using Centrifuge 5430 R (Eppendorf AG, Hamburg, Germany) for 10 min at 17,720× *g*. An aliquot of approximately 60 µL of the supernatant was then taken for UHPLC-MS/MS analysis. In this setup, the dilution factor was 1.5.

#### 3.4.2. Spiked Brain Tissue

For the purpose of method validation, the blank brain tissue was spiked with phytocannabinoids and their metabolites at three concentration levels: 8, 80, and 160 µg/kg of brain tissue (*n* = 5). The required amount of a standard mixture of phytocannabinoids and their metabolites was added to 40 mg of the disintegrated blank brain tissue sample. The sample was thoroughly mixed and incubated for 30 min at room temperature. The preparation of spiked samples was identical to that of the ‘real’ rat brain tissue samples.

### 3.5. Preparation of Matrix-Matched Calibration Standards

A calibration curve was prepared using calibration standards at concentrations of 0.25, 0.5, 1, 2.5, 5, 10, 25, 50, 100, and 250 ng/mL in acetonitrile, which were prepared from a mixture of standards of all phytocannabinoids and their metabolites. Subsequently, each calibration standard was evaporated to dryness under a gentle stream of nitrogen (using the nitrogen concentrator Labicom EVATERM, Olomouc, Czech Republic) and the resulting residue was subsequently reconstituted within an extract of blank brain tissue sample (prepared according to the procedure described in Section 3.4).

### 3.6. Determination of CBD Content in Nanoformulations

To verify the actual CBD content in all nanoformulations used in the pharmacokinetic study, a representative aliquot of each formulation was analyzed using the validated UHPLC-MS/MS method described in Section 3.6. Specifically, 50 µL of each nanoformulation was transferred to a 25 mL volumetric flask, dissolved in ethanol, and filled to the mark with ethanol. The solutions were further diluted 1000-, 100,000-fold with ethanol before analysis.

All samples were spiked with isotopically labeled internal standard (CBD-D_3_) to a final concentration of 30 ng/mL to compensate for potential matrix effects. The analysis was performed in triplicate, and the measured concentrations confirmed the CBD content declared for each carrier system: 10% *w*/*w* for Carrier B, 5% for Carrier C, 4% for Carriers E and F, and 2% for Carrier D. For Carrier A, CBD isolate powder with declared purity of ≥98% was used as reference material.

### 3.7. UHPLC-MS/MS Analysis

The target analysis of phytocannabinoids and their metabolites was performed using a 1290 Infinity UHPLC liquid chromatograph coupled with a 6495 Triple Quadrupole tandem mass spectrometer (Agilent Technologies, Santa Clara, CA, USA).

An Acquity UPLC BEH C18 column (150 × 2.1 mm; 1.7 µm) (Waters Corporation, Milford, MA, USA) maintained at 60 °C was used for the chromatographic separation of phytocannabinoids. Mobile phases consisted of (A) water/acetonitrile (95:5, *v*/*v*) and (B) methanol/acetonitrile/water (60:35:5, *v*/*v*/*v*), both containing 5 mM ammonium formate and 0.1% formic acid. The injection volume was 1 µL. The gradient elution was initiated at 75% mobile phase B (flow rate 0.35 mL/min), followed by a linear increase to 80% B within 0–4 min (flow rate 0.30 mL/min), to 85% B within 4–6 min (flow rate 0.25 mL/min) and to 100% B within 6–15 min (flow rate 0.25 mL/min). Within 15–20 min, the mobile phase B was kept at 100% and the flow rate increased to 0.4 mL/min. The gradient was then terminated by a post time of 3 min at the initial conditions of mobile phase composition and flow rate.

The Dual AJS electrospray ionization (ESI) source with programed polarity switching during the single run was used for the MS/MS analysis combined with dynamic multiple-reaction-monitoring mode (dMRM). Neutral phytocannabinoids and metabolites were analyzed after their ionization in positive mode (formation of protonated ions [M + H]^+^), while phytocannabinoid acids were analyzed after their ionization in negative mode (formation of deprotonated ions [M − H]^−^). The ion source parameters were as follows: gas temperature: 120 °C, gas flow: 11 L/min, nebulizer pressure: 20 psi, sheath gas temperature: 400 °C, sheath gas flow: 12 L/min, capillary voltage: 2000 V and nozzle voltage: 2000 V. iFunnel parameters were as follows: high-pressure RF positive/negative: 130/210 V, low-pressure RF positive/negative: 60/60 V. The MS method parameters were optimized using Agilent MassHunter Optimizer software (Agilent Technologies, Santa Clara, CA, USA).

The analytes were detected and identified based on their specific precursor-to-product ion mass transitions (Supplementary Table S4) and retention times in the samples compared to the matrix-matched calibration standards. To confirm the analyte identity in the sample, at least one quantifier and one qualifier product ion had to be detected with a mass transition relative ratio within ±20% of the mean mass transition ratio detected in the matrix-matched calibration and internal standards. The MassHunter Quantitative Analysis software (version 10.0; Agilent Technologies, Santa Clara, CA, USA) was used to evaluate the obtained UHPLC-MS/MS data. The quantification of the analytes was carried out using a calibration curve composed of at least three concentration levels. For the analytes CBD, ∆^9^-THC, CBG, CBN, ∆^9^-THCA-A, and metabolites, the internal normalization method was used with correction to the internal standards of the deuterated analogs of the relevant analytes. The linearity of the calibration dependencies was checked using the coefficient of determination (*R*^2^), when the value of *R*^2^ would be at least 0.99.

### 3.8. UHPLC-HRMS/MS Analyses

Screening of brain tissue extract composition was performed using an ultra-high-performance liquid chromatography (UHPLC) UltiMate 3000 system Thermo Fisher Scientific (Waltham, MA, USA) equipped with a reverse-phased analytical column Acquity UPLC BEH C18 (150 × 2.1 mm; 1.7 µm). The mobile phase composition, injection volume, and gradient elution were identical to the method for target analysis of phytocannabinoids described in Section 3.7. A Q-Exactive^TM^ Hybrid Quadrupole-Orbitrap^TM^ mass spectrometer from Thermo Fisher Scientific (Waltham, MA, USA) was used for the detection of analytes, and the setup was identical to the one described in our earlier study (Benes et al., 2024) [36]. The mass spectrometer operated in full-scan mode in the 100–1,200 *m*/*z* range followed by parallel reaction monitoring (PRM). Detection conditions were for full scan MS acquisition mode: resolution 35,000 full width at half maximum (FWHM), scan range 100–1,200 *m*/*z*, and for PRM: resolution 17,500 FWHM, and normalized collision energy (NCE) 20, 35, and 40%. Thermo Fisher Scientific’s TraceFinder software (Thermo Fisher Scientific, Waltham, MA, USA) was used for the preliminary identification of matrix-derived compounds such as phospholipids, fatty acids, cholesterol, or preliminary identification of phytocannabinoid metabolites (based on accurate mass measurement and isotopic pattern).

## 4. Conclusions

This study demonstrated that the pharmacokinetic profile of cannabidiol (CBD) in rat brain tissue is strongly dependent on the type of formulation administered. Using a developed and validated UHPLC-MS/MS method, we showed that lipid-based systems, particularly 10% CBD oil (carrier B) and SEDDS with MCT oil (carrier C—self-emulsifying drug delivery system with medium-chain triacylglycerols), enabled rapid and efficient delivery of CBD into brain tissue, as evidenced by the highest *C*_max_ and AUC_0–6_ values. In contrast, proliposomal carriers (E and F) exhibited delayed *T*_max_ and lower *C*_max_, yet maintained prolonged CBD exposure, suggesting their potential for sustained delivery applications. Importantly, high inter-individual variability in brain concentrations was observed across all carriers, underscoring the need to consider formulation strategies that minimize such differences.

In addition to the primary metabolites 7-OH-CBD and 7-COOH-CBD, an unknown compound was detected during their target analysis. Further inspection by high-resolution mass spectrometry (HRMS) confirmed that it was an isomer of OH-CBD.

Together, these results highlight the critical role of formulation in modulating the pharmacokinetics of CBD and support further development of tailored delivery systems for targeted brain exposure. The data also provide a foundation for future studies focused on elucidating the pharmacological relevance of newly identified brain metabolites.

## Figures and Tables

**Table 1:** Average concentrations of CBD (cannabidiol) and its metabolites in brain tissue of experimental animals, calculated for each time point after a single oral dose of 10 mg/kg CBD in different nanoformulations. Carrier A: CBD powder; Carrier B: 10% CBD oil; Carrier C: SEDDS (self-emulsifying drug delivery system) with MCT (Medium-Chain Triglyceride) oil; Carrier D: SEDDS with sesame oil; Carrier E: CBD proliposomes with chitosan coating; Carrier F: CBD proliposomes with chitosan coating prepared by spray drying.

Time (h)	CBD (µg/kg)					
	Carrier A ^1^	Carrier B	Carrier C ^1^	Carrier D	Carrier E	Carrier F
**0.5**	26 ± 10	78 ± 50	50 ± 40	66 ± 67	34 ± 15	24 ± 16
**1**	88 ± 9.2	259 ± 150	226 ± 118	119 ± 43	59 ± 33	51 ± 15
**2**	59 ± 11	191 ± 40	103 ± 73	124 ± 45	57 ± 22	76 ± 52
**4**	24 ± 14	26 ± 15	48 ± 38	34 ± 11	13 ± 6.9	29 ± 2.4
**6**	\	23 ± 22	\	36 ± 27	15 ± 1.5	33 ± 26
**Time (h)**	**7-COOH-CBD (µg/kg)**					
	**Carrier A ^1^**	**Carrier B**	**Carrier C ^1^**	**Carrier D**	**Carrier E**	**Carrier F**
**0.5**	22 ± 0.03	22 ± 5.5	23 ± 10	18 ± 5.2	28 ± 17	16 ± 5.7
**1**	43 ± 6.0	30 ± 10	55 ± 24	50 ± 8.8	27 ± 11	32 ± 23
**2**	35 ± 3.2	49 ± 16	55 ± 22	68 ± 5.6	22 ± 6.7	40 ± 21
**4**	18 ± 3.5	20 ± 3.6	49 ± 46	27 ± 11	14 ± 5.1	47 ± 36
**6**	\	20 ± 11	\	16 ± 16	10 ± 4.1	63 ± 50
**Time (h)**	**7-OH-CBD (µg/kg)**					
	**Carrier A ^1^**	**Carrier B**	**Carrier C ^1^**	**Carrier D**	**Carrier E**	**Carrier F**
**0.5**	8.1 ± 2.1	16 ± 4.7	13 ± 5.5	4.8 ± 3.8	12 ± 3.0	18 ± 6.1
**1**	7.1 ± 5.8	22 ± 1.9	21 ± 4.1	15 ± 5.6	8.1 ± 3.2	6.2 ± 3.8
**2**	<4	8.3 ± 2.5	6.9 ± 0.30	7.9 ± 1.1	7.3 ± 1.6	10 ± 7.3
**4**	<4	<4	<4	<4	<4	<4
**6**	\	<4	\	<4	<4	<4

^1^ For Carriers A and C, the values of the areas under the curve were obtained only for times 0.5, 1, 2, and 4 h after administration.

**Table 2:** Average values for the maximum concentration (*C*max), time to reach the maximum concentration (*T*_max_), and the area under the curve from 0 to 6 h (AUC_0–6_) of CBD (cannabidiol) and its metabolites in rat brain tissue after a single oral dose of 10 mg/kg CBD in different nanoformulations. Carrier A: CBD powder; Carrier B: 10% CBD oil; Carrier C: SEDDS (self-emulsifying drug delivery system) with MCT (Medium-Chain Triglyceride) oil; Carrier D: SEDDS with sesame oil; Carrier E: CBD proliposomes with chitosan coating; Carrier F: CBD proliposomes with chitosan coating prepared by spray drying.

PK Parameter	CBD					
	Carrier A ^1^	Carrier B	Carrier C ^1^	Carrier D	Carrier E	Carrier F
***C*_max_** **(µg/kg)**	88	259	226	124	59	76
***T*_max_** **(h)**	1	1	1	2	1	2
**AUC_0–6_ (µg/kg × h)**	192	596	397	412	186	255
**PK Parameter**	**7-COOH-CBD**					
	**Carrier A ^1^**	**Carrier B**	**Carrier C ^1^**	**Carrier D**	**Carrier E**	**Carrier F**
***C*_max_** **(µg/kg)**	43	30	55	68	27	63
***T*_max_** **(h)**	1	2	1	2	1	6
**AUC_0–6_ (µg/kg × h)**	113	167	184	218	105	250
**PK Parameter**	**7-OH-CBD**					
	**Carrier A ^1^**	**Carrier B**	**Carrier C ^1^**	**Carrier D**	**Carrier E**	**Carrier F**
***C*_max_** **(µg/kg)**	8	22	21	15	17	18
***T*_max_** **(h)**	0.5	1	1	1	0.5	1
**AUC_0–6_ (µg/kg × h)**	9	36	36	25	23	31

^1^ For Carriers A and C, the values of the areas under the curve were obtained only for times 0.5, 1, 2, and 4 h after administration.

## Data Availability

The raw data supporting the conclusions of this article will be made available by the authors on request.

## Supplementary Materials

The following supporting information can be downloaded at https://www.mdpi.com/article/10.3390/molecules30132676/s1, Table S1: Recoveries of phytocannabinoids extracted from rat brain tissue using different extraction solvents; Table S2: Optimized parameters for the determination of phytocannabinoids and their metabolites by QqQ-MS; Table S3: Validation parameters for the quantification of selected phytocannabinoids and their metabolites; Table S4: Mass transitions, collision energy, and retention time of phytocannabinoid metabolites, neutrals, and acids.
