# Supplementary material for: Pharmacokinetics of Cannabidiol in Rat Brain Tissue After Single-Dose Administration of Different Formulations
Source: Molecules. 2025 Jun 20;30(13):2676. doi: 10.3390/molecules30132676 (PMC12250628; doi:10.3390/molecules30132676)
Supplement: Supplementary file 1 [file molecules-30-02676-s001.zip › molecules-3663097-supplementary.pdf]

# Pharmacokinetics of Cannabidiol in Rat Brain Tissue After Single-Dose Administration of Different Formulations

**Zuzana Binova[1], Frantisek Benes[1], Marie Zlechovcova[1], Matej Maly[1], Petr Kastanek[2], Monika Cahova[3], Milena Stranska[1] and Jana Hajslova[1,*]**

[1] Department of Food Analysis and Nutrition, University of Chemistry and Technology, Technická 3, 166 28 Prague, Czech Republic; zuzana.binova@vscht.cz (Z.B.); frantisek.benes@vscht.cz (F.B.); marie.zlechovcova@vscht.cz (M.Z.); matej.maly@vscht.cz (M.M.); milena.stranska@vscht.cz (M.S.)

[2] Department of Biotechnology, University of Chemistry and Technology Prague, Technická 5, 166 28 Prague 6, Czech Republic; petr.kastanek@vscht.cz

[3] Institute for Clinical and Experimental Medicine, Vídeňská 1958, 140 21 Prague 4, Czech Republic; moca@ikem.cz

Table S1. Recoveries (%) of various phytocannabinoids extracted from rat brain tissue evaluated using different solvent systems (acetonitrile, isopropyl alcohol, methanol and ethanol), modified acetonitrile-based extractions (with water ratios of 3:1 and 30:1) and a QuEChERS-based extraction method.

| Extraction/Analytes | Recoveries (%) | | | | | | | | | | | | |
|---|---|---|---|---|---|---|---|---|---|---|---|---|---|
| | CBD | Δ9-THC | CBDV | CBDVA | THCV | CBN | THCVA | CBNA | CBL | CBC | CBLA | CBDA | CBGA |
| **Acetonitrile** | 110 | 94 | | | | | | | | | | | |
| **Isopropyl alcohol** | 24 | 97 | | | | | | | | | | | |
| **Methanol** | 70 | 111 | | | | | | | | | | | |
| **Ethanol** | 81 | 172 | | | | | | | | | | | |
| **Acetonitrile (3:1)** | 90 | 85 | 69 | 82 | 83 | 72 | 80 | 192 | 67 | 83 | 69 | 30 | 64 |
| **Acetonitrile (30:1)** | 110 | 82 | 94 | 77 | 82 | 69 | 82 | 114 | 54 | 40 | 88 | 53 | 69 |
| **QuEChERS** | 100 | 98 | 108 | 116 | 122 | 114 | 111 | 109 | 94 | 127 | 117 | 19 | 101 |

Table S2. Optimized parameters for the determination of phytocannabinoids and their metabolites by QqQ-MS.

| Parameter Types | Start Value | End Value | Step Value |
|---|---|---|---|
| **High Pressure RF (V)** | 70 | 210 | 20 |
| **Low Pressure RF (V)** | 40 | 160 | 20 |
| **Sheath Gas Temperature (°C)** | 200 | 400 | 50 |
| **Sheath Gas Flow (L/min)** | 10 | 12 | 1 |
| **Drying Gas Temperature (°C)** | 120 | 230 | 30 |
| **Drying Gas Flow (L/min)** | 11 | 20 | 2 |
| **Nebulizer Pressure (psi)** | 20 | 40 | 5 |
| **Capillary Voltage (V)** | 1500 | 4500 | 500 |
| **Nozzle Voltage (V)** | 0 | 2000 | 500 |

Table S3. Validation parameters.

| Compounds | Rat brain tissue spiked with a mixed solution of all targeted analytes | | | | | | | | | LOQ (µg/kg)[1] | Linear range (µg/kg) |
|---|---|---|---|---|---|---|---|---|---|---|---|
| | 8 µg/kg brain, n=5 | | | 80 µg/kg brain, n=5 | | | 160 µg/kg brain, n=5 | | | | |
| | Recoveries (%) | RSD (%) | SSE (%) | Recoveries (%) | RSD (%) | SSE (%) | Recoveries (%) | RSD (%) | SSE (%) | | |
| **Neutral phytocannabinoids** | | | | | | | | | | | |
| **CBGV** | 97 | 10 | 110 | 110 | 12 | 108 | 104 | 12 | 110 | 4 | 4 - 160 |
| **CBDV** | 94 | 7 | 110 | 97 | 5 | 125 | 89 | 6 | 98 | 1.6 | 1.6 - 400 |
| **CBD** | 108 | 7 | 152 | 95 | 5 | 107 | 99 | 1 | 83 | 4 | 4 - 400 |
| **CBG** | 94 | 5 | 103 | 96 | 4 | 116 | 87 | 12 | 102 | 1.6 | 1.6 - 400 |
| **THCV** | 86 | 7 | 109 | 88 | 5 | 133 | 82 | 6 | 106 | 1.6 | 1.6 - 400 |
| **CBN** | 94 | 1 | 114 | 92 | 1 | 114 | 95 | 1 | 83 | 1.6 | 1.6 - 400 |
| **$\Delta^9$-THC** | 82 | 3 | 107 | 85 | 2 | 114 | 90 | 2 | 85 | 1.6 | 1.6 - 400 |
| **$\Delta^8$-THC** | 90 | 4 | 99 | 96 | 7 | 115 | 85 | 7 | 96 | 1.6 | 1.6 - 400 |
| **CBL** | 77 | 4 | 93 | 84 | 9 | 111 | 71 | 7 | 95 | 1.6 | 1.6 - 400 |
| **CBC** | 81 | 5 | 157 | 86 | 7 | 160 | 77 | 2 | 146 | 4 | 4 - 160 |
| **Phytocannabinoid acids** | | | | | | | | | | | |
| **CBDVA** | 82 | 7 | 80 | 80 | 4 | 106 | 73 | 7 | 89 | 4 | 4 - 160 |
| **CBDA** | 71 | 3 | 87 | 79 | 5 | 94 | 80 | 13 | 103 | 1.6 | 1.6 - 160 |
| **CBGA** | 83 | 2 | 129 | 92 | 6 | 116 | 84 | 8 | 107 | 1.6 | 1.6 - 400 |
| **THCVA** | 81 | 3 | 100 | 77 | 4 | 115 | 73 | 4 | 102 | 4 | 4 - 400 |
| **CBNA** | 86 | 4 | 156 | 93 | 8 | 168 | 83 | 10 | 153 | 1.6 | 1.6 - 400 |
| **$\Delta^9$-THCA-A** | 85 | 5 | 102 | 72 | 6 | 284 | 73 | 4 | 290 | 1.6 | 1.6 - 160 |
| **CBLA** | 95 | 3 | 156 | 102 | 6 | 280 | 83 | 6 | 238 | 1.6 | 1.6 - 160 |
| **CBCA** | 69 | 7 | 186 | 86 | 14 | 261 | 67 | 7 | 217 | 4 | 4 - 160 |
| **Metabolites of phytocannabinoids** | | | | | | | | | | | |
| **7-COOH-CBD** | 112 | 6 | 127 | 84 | 6 | 107 | 88 | 7 | 97 | 8 | 8 - 160 |
| **7-OH-CBD** | 96 | 1 | 97 | 94 | 4 | 96 | 93 | 6 | 94 | 8 | 8 - 160 |
| **11-nor-9-COOH-$\Delta^9$-THC-gluc** | 61 | 9 | 80 | 70 | 20 | 68 | 61 | 10 | 59 | 4 | 4 - 160 |
| **11-OH-$\Delta^9$-THC** | 93 | 4 | 89 | 92 | 4 | 86 | 98 | 2 | 85 | 4 | 4 - 400 |
| **11-nor-9-COOH-$\Delta^9$-THC** | 83 | 2 | 86 | 80 | 8 | 98 | 84 | 7 | 109 | 8 | 8 - 400 |

[1]In this study, the limit of detection (LOD) was determined to be one-third of the limit of quantification (LOQ)

Table S4. Mass transitions, collision energy and retention time of phytocannabinoid metabolites, neutrals and acids.

| Compound Name | Molecular Formula | Ret Time (min) | Polarity | Precursor Ion (*m/z*) | Collision Energy (eV) | Product Ion (*m/z*)[1] |
|---|---|---|---|---|---|---|
| **Neutral phytocannabinoids** | | | | | | |
| **CBGV** | $C_{19}H_{28}O_2$ | 3.5 | $[M+H]^+$ | 317.2 | 14/34/50 | 165.1/123.0/91.0 |
| **CBDV** | $C_{19}H_{26}O_2$ | 4.3 | $[M+H]^+$ | 287.2 | 24/16/16 | 165.0/135.2/231.0 |
| **CBD** | $C_{21}H_{30}O_2$ | 4.9 | $[M+H]^+$ | 315.2 | 28/40/20 | 193.0/123.0/135.1 |
| **CBD-D$_3$** | $C_{21}H_{27}O_2D_3$ | 4.9 | $[M+H]^+$ | 318.3 | 24/20/28 | 196.0/135.1/107.2 |
| **CBG** | $C_{21}H_{32}O_2$ | 5.0 | $[M+H]^+$ | 317.2 | 20/40/36 | 193.0/123.0/137.0 |
| **CBG-D$_3$** | $C_{21}H_{29}O_2D_3$ | 5.0 | $[M+H]^+$ | 320.3 | 22/42/46 | 196.1/123.1/69.1 |
| **THCV** | $C_{19}H_{26}O_2$ | 5.3 | $[M+H]^+$ | 287.2 | 24/20/40 | 165.0/135.1/123.1 |
| **CBN** | $C_{21}H_{26}O_2$ | 7.5 | $[M+H]^+$ | 311.2 | 20/16/16 | 223.1/293.2/241.1 |
| **CBN-D$_3$** | $C_{21}H_{23}O_2D_3$ | 7.5 | $[M+H]^+$ | 314.2 | 26/18/22 | 223.1/296.2/241.1 |
| **$\Delta^9$-THC** | $C_{21}H_{30}O_2$ | 8.6 | $[M+H]^+$ | 315.2 | 36/24/32 | 123.0/135.0/107.2 |
| **$\Delta^9$-THC-D$_3$** | $C_{21}H_{27}O_2D_3$ | 8.6 | $[M+H]^+$ | 318.3 | 24/40/24 | 196.0/123.0/135.1 |
| **$\Delta^8$-THC** | $C_{21}H_{30}O_2$ | 9.1 | $[M+H]^+$ | 315.2 | 24/36/20 | 193.0/123.1/259.1 |
| **CBL** | $C_{21}H_{30}O_2$ | 9.4 | $[M+H]^+$ | 315.2 | 20/44/28 | 235.2/81.1/165.1 |
| **CBC** | $C_{21}H_{30}O_2$ | 10.4 | $[M+H]^+$ | 315.2 | 16/32/16 | 259.1/123.0/233.1 |
| **Phytocannabinoid acids** | | | | | | |
| **CBDVA** | $C_{20}H_{26}O_4$ | 3.5 | $[M-H]^-$ | 329.2 | 20/32/24 | 311.2/217.2/283.1 |
| **CBDA** | $C_{22}H_{30}O_4$ | 5.1 | $[M-H]^-$ | 357.2 | 24/24/24 | 339.2/311.2/289.2 |
| **CBGA** | $C_{22}H_{32}O_4$ | 5.9 | $[M-H]^-$ | 359.2 | 20/24/40 | 341.3/315.2/191.1 |
| **THCVA** | $C_{20}H_{26}O_4$ | 8.0 | $[M-H]^-$ | 329.2 | 24/28/36 | 285.2/217.1/163.1 |
| **CBNA** | $C_{22}H_{26}O_4$ | 9.2 | $[M-H]^-$ | 353.2 | 20/44/64 | 309.3/279.1/222.0 |
| **$\Delta^9$-THCA-A** | $C_{22}H_{30}O_4$ | 11.2 | $[M-H]^-$ | 357.2 | 28/36/32 | 313.1/245.2/311.3 |
| **$\Delta^9$-THCA-A-D$_3$** | $C_{22}H_{27}O_4D_3$ | 11.2 | $[M-H]^-$ | 360.2 | 26/34/38 | 316.2/248.2/194.1 |
| **CBCA** | $C_{22}H_{30}O_4$ | 11.6 | $[M-H]^-$ | 357.2 | 24/28/44 | 313.2/339.2/191.1 |
| **CBLA** | $C_{22}H_{30}O_4$ | 12.0 | $[M-H]^-$ | 357.2 | 24/44/28 | 313.2/191.1/339.2 |
| **Metabolites of phytocannabinoids** | | | | | | |
| **7-COOH-CBD** | $C_{21}H_{28}O_4$ | 2.0 | $[M+H]^+$ | 345.2 | 18/18/30 | 327.2/299.2/193.1 |
| **7-COOH-CBD-D$_3$** | $C_{21}H_{25}O_4D_3$ | 2.0 | $[M+H]^+$ | 348.2 | 22/20/22 | 302.4/196.1/274.1 |
| **7-OH-CBD** | $C_{21}H_{30}O_3$ | 2.1 | $[M+H]^+$ | 331.2 | 14/26/20 | 313.0/201.1/193.1 |
| **7-OH-CBD$_3$** | $C_{21}H_{27}O_3D_3$ | 2.1 | $[M+H]^+$ | 334.3 | 22/22/26 | 316.1/201.0/133.1 |
| **11-nor-9-COOH-$\Delta^9$-THC-glu** | $C_{27}H_{36}O_{10}$ | 2.5 | $[M+H]^+$ | 521.2 | 16/26/52 | 345.2/327.2/193.1 |
| **11-nor-9-carbo-$\Delta^9$-THC-glu-D$_3$** | $C_{27}H_{33}O_{10}D_3$ | 2.5 | $[M+H]^+$ | 524.3 | 14/30/38 | 348.2/330.2/302.2 |
| **11-hydroxy-$\Delta^9$-THC** | $C_{21}H_{30}O_3$ | 4.1 | $[M+H]^+$ | 331.2 | 18/20/32 | 313.1/193.1/133.1 |
| **11-hydroxy-$\Delta^9$-THC-D$_3$** | $C_{21}H_{27}O_3D_3$ | 4.1 | $[M+H]^+$ | 334.2 | 12/32/24 | 316.1/196.1/133.1 |
| **11-nor-9-carboxy-$\Delta^9$-THC** | $C_{21}H_{28}O_4$ | 4.6 | $[M+H]^+$ | 345.2 | 14/22/30 | 327.2/299.2/193.1 |
| **11-nor-9-carboxy-$\Delta^9$-THC-D$_9$** | $C_{21}H_{19}O_4D_9$ | 4.6 | $[M+H]^+$ | 354.3 | 18/20/34 | 336.3/308.3/124.1 |

[1]The product ions are listed according to their abundance in the MS/MS spectrum, with the first one listed being the quantifier ion and the others the qualifier ions.
